# Possible Risk Factors for Severe Anemia in Hospitalized Sickle Cell Patients at Muhimbili National Hospital, Tanzania: Protocol for a Cross-Sectional Study

**DOI:** 10.2196/resprot.7349

**Published:** 2018-02-28

**Authors:** Furahini Tluway, Florence Urio, Bruno Mmbando, Raphael Zozimus Sangeda, Abel Makubi, Julie Makani

**Affiliations:** ^1^ Department of Hematology and Blood Transfusion Muhimbili University of Health and Allied Sciences Dar es Salaam United Republic Of Tanzania; ^2^ Department of Biochemistry University of Health and Allied Sciences Dar es Salaam United Republic Of Tanzania; ^3^ Tanga Research Centre National Institute for Medical Research Tanga United Republic Of Tanzania; ^4^ Department of Pharmaceutical Microbiology Muhimmbili University of Health and Allied Sciences Dar es Salaam United Republic Of Tanzania; ^5^ Bugando Medical Centre Mwanza United Republic Of Tanzania

**Keywords:** severe anemia, sickle cell, hemoglobin, hospital-based surveillance, protocol, Tanzania

## Abstract

**Background:**

Sickle cell disease (SCD) is the most common inherited disorder worldwide, with the highest burden in sub-Saharan Africa. The natural history of SCD is characterized by periods of steady state interspersed by acute episodes. The acute anemic crises may be transient and are precipitated by treatable factors like infections, nutritional deficiencies, and sequestration. Anemia is almost always present, although it occurs at different levels of severity.

**Objective:**

This paper describes the protocol of a cross-sectional study to determine the prevalence of severe anemia and associated factors among sickle cell patients hospitalized at the Muhimbili National Hospital.

**Methods:**

This is an ongoing, descriptive, cross-sectional, hospital-based study among individuals with SCD, admitted to the Muhimbili National Hospital in Dares Salaam, Tanzania. A minimum sample size of 369 was calculated based on the previous prevalence of hospitalizations due to severe anemia (20%) in the same cohort. We are using a piloted standardized case report form to document clinical and laboratory parameters following informed consent. Data analysis will be performed using Stata software. Severe anemia is defined as Hb<5g/dL. Chi-square or Fisher’s exact test will be used to ascertain association between categorical variables, and *t*-test will be used for numerical variables. Regression models for severe anemia against explanatory and confounding variables will be run, and results will be presented as adjusted odds ratio with 95% confidence intervals. A *P* value of <.05 will be considered significant.

**Results:**

Enrolment commenced in January 2015 and concluded in September 2016. Complete data analysis will begin in February 2018. The study results are expected to be published in May 2018.

**Conclusions:**

This protocol paper will provide a useful and practical model for conducting cross-sectional studies in hospitalized patients that cover a wide ranging of clinical and laboratory variables.

## Introduction

Sickle cell disease (SCD) is the most common inherited disorder worldwide with the highest burden in sub-Saharan Africa [1]. It is also estimated that between 7800 and 11,000 children with SCD are born in Tanzania every year [2]. Most of these children will not be diagnosed as there are no neonatal screening programs in place, and up to 50% of these children will die before the age of 5 years [1]. It is estimated that 6 million people would be living with SCD in Africa if the average survival of affected children reaches half the African norm [3]. With demographic transition, the survival of SCD beyond childhood is increasing. The survivors will suffer from chronic ill health due to a number of factors including anemia [4].

Anemia is a condition in which the number of red blood cells (and consequently their oxygen-carrying capacity) is insufficient to meet the body’s physiological needs. These needs vary with a person’s age, sex, residential elevation above sea level (altitude), smoking behavior, and different stages of pregnancy [5]. The World Health Organization has defined very severe anemia as hemoglobin (Hb) levels of <5 g/dL. The progression of SCD is characterized by periods of dormancy coupled with periods of acute crises. Usually transient, the acute anemic crises may be precipitated by treatable causes. In SCD, severe anemia (ie, low hemoglobin, Hb <5 g/dL [3.8 (CI 1.8–8.2); *p*=.001]) has been shown to be an independent predictor of death [6]. Similarly, a study by Calis and colleagues from Malawi indicated a mortality rate of three folds (95% CI 1.3- 6.9) in SCD individuals with severe anemia compared to those with nonsevere anemia [7].

The major contributors of severe anemia in SCD in Africa are not known. Studies on African children on this topic have reported an association with bacteraemia, malaria, hookworm, HIV, as well as deficiency in vitamins A, B12 and glucose-6-phosphatase dehydrogenase [8-11,12]. Nutritional deficiencies in children with SCD may be due to poor dietary intake and increased requirements [13]. In endemic areas, malaria has been associated with severe anemia and is a major cause of morbidity and mortality [12,14-16]. Hyperhemolysis, sequestration transient red cell aplasia [17] and bacteremia [18], have been implicated in causing severe anemia in SCD cases. Hyperhemolysis may be an independent complication of SCD [19] or may be due to infections (eg, malaria and bacteremia), hemolytic transfusion reaction or drugs. Acute splenic sequestration is a major cause of death although this is often idiopathic [20-22].

Blood transfusion is almost always used to manage severe anemia in SCD [23]. There are, however, concerns for blood transfusion, particularly in Africa, where the risk of transmitting infections including HIV, and hepatitis B and C is still high [24,25]. With multiple transfusions, patients are at risk of hemolytic transfusion reactions, alloimmunization [26-28] and iron overload [23,29]. It is clear that blood supply is inadequate and costly although the blood transfusion requirements for SCD are not known [27].

Although the burden and factors associated with severe anemia have been described in children and pregnant women in sub-Saharan Africa [6,8,11,30,31], detailed characterization of these factors in SCD individuals is lacking. This study, therefore, aims to establish the prevalence and spectrum of factors associated with severe anemia in SCD. The information obtained from this study has the potential to significantly impact the clinical course and survival of SCD individuals in Tanzania.

## Methods

### Study Design and Site

This is a hospital-based, descriptive, cross-sectional study among SCD individuals hospitalized at the Muhimbili National Hospital (MNH). MNH is the national referral hospital in collaboration with Muhimbili University of Health and Allied Sciences, which is the oldest and largest biomedical university in Tanzania. Muhimbili is located in Dar es Salaam on the eastern coast of Tanzania.

### Study Population

The study population consists of individuals with SCD who are already registered in the Muhimbili Sickle Cohort (MSC) and are hospitalized at MNH. The MSC has enrolled 5430 individuals with SCD between 2004 and March 2016. Of these 51.5% and 48.5% are male and female, respectively. The majority (65%) are below the age of 18 years. In this study, we enrolled hospitalized patients who consented and fulfilled inclusion criteria during the 21-monthstudy period (January 2015 to September 2016).

### Sample Size Determination

The sample size for this study was estimated to be 369 patients and it was based on the finite population of 800 SCD cases (ie, number of annual SCD hospitalization, from 2014 estimates). This sample has a power of 80% to detect the prevalence of severe anemia of 20% with a 4% margin of error and confidence interval of 95%.

### Eligibility criteria

The following inclusion and exclusion criteria were used:

Inclusion criteria

- Confirmed SCD; HbSS by High Performance Liquid Chromatography or Hemoglobin Electrophoresis
- Enrolled in the MSC
- Hospitalization at MNH during the study period

Exclusion criteria

- Individuals on hydroxyurea
- Blood transfusion in the past 4 weeks
- Readmission within the past 4 weeks

### Procedures During Hospitalization

**Figure 1:**
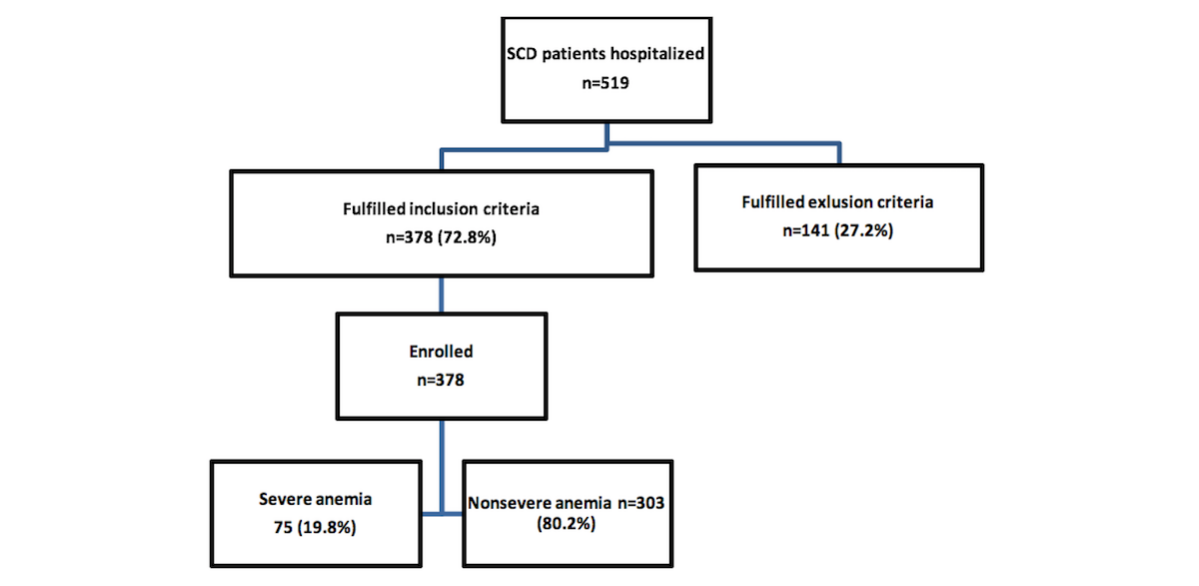
Enrolment flow chart. SCD: sickle cell disease.

The enrolment procedure is illustrated in Figure 1. Clinical surveillance of the adult and pediatric admitting wards to identify individuals admitted with known SCD was maintained throughout the study period. On arrival, SCD patients were admitted following normal hospital procedures.

A general examination was performed that included a record of temperature, weight, blood pressure, peripheral oxygen saturation, pulse, and respiratory rate. Detailed clinical history and physical examination were undertaken to determine the events leading to hospitalization. The study procedures were explained to the patients as well. Details of tests to be performed and samples needed were given to the patients by the study clinician. HIV pretesting counseling was done to all eligible patients. Consent was sought after all the information had been given to the patients.

### Patient Care and Management

Confirmed SCD individuals admitted and enrolled were managed according to the standardized guidelines on the management of SCD. Additional investigations were requested depending on clinical indications. Clinical outcomes and laboratory results (Textbox 1) of every patient were documented in the hospital case file as well as in the study’s case report form (CRF). The results of all the tests done were made known to the patient and the attending doctors to guide patient management and care. We routinely conducted HIV postcounseling to all HIV positive patients, and upon discharge they were referred to the HIV Care and Treatment Centers near them.

### Procedures for Collection of Samples

Ten milliliters of blood was collected from all participants under aseptic technique. Of these, 2 mL of blood was collected in a tube with ethylenediamine tetraacetic acid (EDTA) anticoagulant, 2 red tops (serum separator) were filled with 2 mL of blood each, and 4 mL was taken for blood culture. Samples for blood culture were collected in commercially prepared BacTec bottles. In addition, stool and urine samples were collected in empty sterile bottles. All samples were labelled with patients' name, SCD identification number and a special ID number created for the purposes of this study.

### Processing and Separation of Samples

Of the EDTA sample, 5µl was used to perform a malaria rapid test (RDT). The remaining sample was used to perform a full blood count. This includes a peripheral blood cell count, both total and differential white cell count, as well as red blood cell indices. These tests were performed immediately after sample collection.

For the serum separated samples, clinical chemistry analysis as well as micronutrient analysis (iron, folate, retinol and B12) were performed on the batched serum samples after enrolment had concluded.

### Procedures for Laboratory Investigations

#### Parasitology

##### Malaria Rapid Test

Venous blood in a EDTA tube or capillary blood sample via puncture of a finger was required for the malaria test. The malaria device Pf (HRP2) Ag RDT was used and 5µl of blood was applied to the sample using a pipette or micropipette. 60 µl of assay buffer solution (2 drops of the bottle type) was put into the A well. Results were read in 20 minutes. Correction for white cell count was conducted for counting parasite density in thick films. If the test could not be performed immediately, the blood was stored at 2-8˚C for 3 days. All test components were performed at room temperature.

Quality control was used to assure that the malaria Pf cassette test was working properly and that the results being generated are correct. If no lines were visible, or if no control line (C) formed, whether or not a sample line (T) appeared, the assay was deemed invalid and was repeated with a new sample and cassette.

##### Microscopy for Malaria Parasitaemia

Thick films for malaria diagnosis was prepared from the blood sample at the time of collection or from the EDTA bottle in the laboratory.

**Key variables.**

Outcome variable: hemoglobin levels (categorical: Hb <5 g/dL or ≥5 g/dL)

Independent variables

Sociodemographic characteristics

Ethnicity

Residency

Age as continuous and as categorical variable (<18 or ≥18 years)

Sex (categorical: male or female)

Symptoms and signs

Pallor (categorical: Yes [Y] or No [N])

Jaundice (categorical: Y/N), if yes (categorical: tinge, moderate, severe)

O_2_ saturation-continuous, hypoxia (categorical: <95% or ≥95%)

Enlarged spleen (categorical: Y/N), size (continuous)

Enlarged liver (categorical: Y/N), size (continuous)

Past medical history

Admissions in the past 12 months (categorical: Y/N), frequency (numerical)

Blood transfusion in the past 12 months (categorical: Y/N), frequency (continuous), units (continuous)

Pain in the past 12 months (categorical: Y/N), frequency (continuous)

Stroke in the past 12 months (categorical: Y/N)

History of heart failure (categorical: Y/N)

Laboratory variables (continuous and categorical); see Table 1)

Hematological: full blood count and reticulocyte count

Parasitology: malaria and hookworm

Microbiology: bacteraemia and HIV infection

Biochemistry: liver and renal function tests, lactate dehydrogenase, C reactive protein, serum folate, vitamin B12, vitamin A and serum iron studies

Thick films were stained using Giemsa staining and examined by microscopy for the presence of malaria parasites using standard methods. The number of parasites (trophozoites) was counted against 200 white blood cells. A slide was reported negative for malaria parasites after at least 100 high-powered microscopic fields were examined. Counting malaria parasites against 200 white blood cells may have resulted in underestimating malaria density as the white cell count in SCD patients tends to be higher due to inflammation and hematology analyzers counting nucleated red blood cells as neutrophils and small lymphocytes. Therefore, ideally, the white cell count should be corrected and “normal” reference values for SCD and non-SCD individuals be calculated and used.

The quality assurance process involved the following: all positive slides were read by a second person. If there were discrepancies between the two readings, then a third person was asked to intervene. For the negative slides, one out of every ten slides was read by a second person **.**

##### Hookworm

Fresh stool was collected in a clean container to test for hookworm infection. A portion of fresh stool was taken with a spatula or wooden applicator and placed in a test tube, and then the test tube was filled with normal saline. The mixture was mixed well and centrifuged for 5 min at 3000 rpm (Rotina 46, Germany). The supernatant was discarded and with help of a Pasteur pipette, the deposit (stool) was extracted and a small drop was placed on a slide. The deposit on the slide was covered by the cover slip and immediately observed for the hookworm ova under the microscope at 10x magnification. The counting was performed 20 minutes after collection. This method allows for other egg species to be identified **.**

We ensured that samples were collected in a clean container so as to avoid any false positive results. A picture of hookworm ova was available to make sure of correct diagnosis.

##### Hematology

The Sysmex XT2000i–Hematology automated analyzer was used to obtain the full blood count. The machine gives a wide range of hematological indices such as white blood cell count, reticulocyte count, hemoglobin levels and platelet count. 2µl of nonclotted venous blood was collected in EDTA tubes (purple caps). The samples were arranged on the roller mixer ready to be tested. Alternatively, the sample was inverted gently end to end (approximately eight times) until the cell bottom of the tube was completely suspended.

On the standard tools bar, the manual section was selected, the patient details were entered and the enter button was pressed. Then the sample vial was held to the needle in front of the start button, and the start button pressed. Once the analysis was done, the results were printed out and reviewed for any panic values, which were then reported to the clinician immediately. If the differential values were missing from the printed results, the sample was diluted in a ratio of 1:5 using the cell pack reagent and the percentage values obtained. After the sample had been analyzed, plasma and buffy coat were obtained and stored under –20°C. This was achieved by centrifuging the EDTA blood at 3000 rpm for 10 minutes, thereafter separating the plasma and buffy coat in graduated cryo-preservative tubes.

The controls for Sysmex machine XT2000i were commercially prepared reagents in 3bottles. Tube one contained low ranges of the hematological indices, tube two for normal and tube three for high hematological values. The quality control procedure was performed before running the samples. The controls needed to be at room temperature before the quality control procedure. The sampler mode from the main menu screen was selected and the tubes were arranged on the sample loader tray with their barcode facing the red barcode light. Then the start button was clicked for the analyzer to start analyzing the controls. Once done, one researcher ensured that the controls had been run on the analyzer and that they were within the acceptable limits.

##### Chemistry

Architect Kits was used for aspartate aminotransferase (AST), alanine aminotransferase (ALT), creatinine, lactate dehydrogenase (LDH), vitamin B12, folate and C reactive protein (CRP). The Retinol Kit was used for retinol binding protein (vitamin A). The analyzer used was Architect Ci 4100, which includes the chemistry analyzer C4000 (for AST, ALT, creatinine, LDH and CRP2) and the immunoassay analyzer i1000SR (for vitamin B12 and folate). Our samples were stored at –80ºC for a maximum of 90 days.

The batched samples that have been collected throughout the study period will be assayed using a chemistry analyzer (Roche Cobas Mira, New York, USA or Abbott Architect, New York, USA) for the following parameters: bilirubin (total and direct); LDH), iron studies (serum ferritin, serum iron and transferrin saturation), CRP, folate, AST, ALT, vitamin A (retinol) and vitamin B12. Quality assurance was done using commercially prepared reagents that are run on a daily basis with the readings documented following laboratory protocol.

#### Microbiology

##### Blood Cultures

Blood cultures were processed using standard hospital laboratory procedures. Culture media were prepared in a microbiological laboratory and identification followed conventional techniques.

##### Blood Culture Methodology

Samples were drawn from patients, stored in the BacTec blood culture bottles, and inserted directly into the instrument as soon as possible to ensure performance efficacy. Before placing vials into the instrument, barcodes were scanned and placed in their assigned stations through the vial entry activity. When scanning barcodes, the vial was placed in the alignment block in front of the scanner with the barcode label facing the scanner. The vial was rotated slightly so that the scanner could read the label. A single beep indicated a successful (good) scan. Station assignments were calculated by the system software to balance the rotor. In order to maintain rotor balance, vials were introduced and placed where the system indicated.

The vial was carefully pushed into the station. In order to ensure that the vial was fully seated in the station, the shoulders were pressed. The vials were not twisted or turned once they were placed in the stations. Vials were not removed except in the following conditions:

- Removal of positive
- Removal of negative
- Identification of anonymous vials

When microorganisms are present, they metabolize nutrients in the culture medium, releasing carbon dioxide, which reacts with a dye in the sensor. The dye modulates the amount of light that is absorbed by a fluorescent material in the sensor. The instrument’s photo detectors then measure the level of fluorescence, which corresponds to the amount of carbon dioxide released by any micro-organisms present. This measurement is interpreted by the system in accordance with the preprogrammed positivity parameters.

#### Sample Handling

##### Positives

Many positive cultures were detected in the first 24 hours after inoculation. Subculturing the positive cultures was done in blood agar, chocolate agar, MacConkey agar, and in other culture media as appropriate. Gram staining was performed for each positive vial. Preliminary antimicrobial susceptibility and identification procedures were performed from fluid in the culture vials.

##### Negatives

Ongoing negative vials were kept for 7 days before being discarded as negatives.

#### HIV Test: Rapid Test (Determine and Unigold)

Serum/whole blood samples were used for this test. The samples were centrifuged at 3000 rpm for 10 minutes. Thereafter, the serum was graduated 1.25 mL tubes for the HIV test. With whole blood samples, the blood was directly tested for HIV test. The test was performed according to the Tanzania National HIV Rapid Test Algorithm.

### Data Management

#### Data Collection

All clinical and laboratory information was collected using a preformatted CRF that was designed specifically for this study. This was piloted for a period of 2 weeks and adjusted accordingly. The CRF was reviewed weekly for the first month of data collection to amend variables and improve the quality of data collected. Tools for collecting anthropometric measurements and vital signs were calibrated according to manufacturer’s specifications. All data will be linked to the MSC database, a Web-based system known as MySQL (Sun Microsystems Inc, Santa Clara, California, USA).

Laboratory data is being collected from different sources. For hematology data, the results were printed from hematology analyzers. Results generated from all laboratories were photocopied and the copies were added to the patient’s case notes. The original copies of results were attached to the corresponding CRFs.

#### Data Cleaning

Data verification and cleaning is ongoing following double entry, and inconsistencies are being corrected. Missing data, not obtained during the data collection period, will be collected during subsequent visits or by telephone.

#### Data Security

The CRFs are stored in filing cabinets and locked, with access only to personnel involved in the study and to a few key personnel who are authorized by the principal investigator. Data backup is done on a daily basis onto 2 storage devices and stored in 2 different physical locations.

#### Analysis Plan

Data is double entered and validated in a software application developed using MySQL database and PHP, a server-side HTML scripting language.

### Statistical Methods

Descriptive statistics will be summarized using cross-tabulation and frequencies for categorical data. For continuous variables, measures of central tendencies will be used after checking for normality and transformation where necessary. All estimates will be presented with 95% confidence intervals. Proportions will be compared using Chi-square test of Fisher’s exact test for categorical variables, while continuous variables will be compared using *t*-test or analysis of variance. These will be adjusted for age and sex. Logistic and linear regression models will be used in assessment of the risk factors for severe anemia for the binary and continuous variables, respectively, as well as for controlling for confounders.

Modelling procedures will start with the univariate models where the response variable will be fitted against each explanatory variable. Only sets of explanatory variables with a *p* value <.10 will be included in the multivariate models. Linear and nonlinear relationship between response and explanatory variables as well as the interaction between the explanatory variables and how these change in relation to age groups and sex *,* will be explored during model fitting. Both forward and backward elimination methods will be used to decide which variables remain in the minimal model based on the likelihood ratio test. We will produce a single, final model combining both laboratory and clinical variables as this will be the best model predicting severe anemia. Data analysis will be performed using Stata software (version 11) and R (version 3.3.2).

Table 1 describes the laboratory variables; these will be compared among those with and without severe anemia.

For univariate and multivariate analyses, it will depend on how the *p* values look like after analysis. We will decide which clinical and laboratory variables are most important and should be included; these may be the most important factors from literature and/or have enough participants in comparison groups to allow for reasonable comparison.

**Table 1:** Baseline laboratory characteristics of the study population.

Variable	Variable type	Measure of central tendency	Categories
Hemoglobin (g/dL)	Numerical, categorical	Mean ± SD	<5, ≥5
Mean corpuscular volume (fl)	Numerical, categorical	Mean ± SD	<80, 80-96, >96
Mean corpuscular hemoglobin (pg)	Numerical, categorical	Mean ± SD	<27, 27-33, >33
Reticulocyte count (%)	Numerical, categorical	Mean ± SD	<0.5, 0.5-1.5, >1.5
White blood count x 10³/L	Categorical	Mean ± SD	≥11, <11
Malaria	Categorical		Positive, negative
HIV	Categorical		Positive, negative
Stool hookworm	Categorical		Positive, negative
Alanine aminotransferase (U/L)	Numerical, categorical	Mean ± SD	<17, 17-63, >63
Aspartate aminotransferase (U/L)	Numerical, categorical	Mean ± SD	<18, 18-40, >40
Serum creatinine (µmol/L)	Numerical, categorical	Mean ± SD	<62, 62-115, >115
Folate (ng/mL)	Numerical, categorical	Mean ± SD	< 2.6, ≥ 2.6
Retinol (µg/L)	Numerical, categorical	Mean ± SD	<30, 30-80, >80
Vitamin B12 (pg/mL)	Numerical, categorical	Mean ± SD	< 187, ≥187
C reactive protein (mg/L)	Numerical, categorical	Mean ± SD	<8, ≥8
Lactate dehydrogenase (U/L)	Numerical, categorical	Mean ± SD	<50, 50-200, >200
Bacteraemia	Categorical		Yes/no, type, sensitivity
Serum ferritin (ng/mL)	Numerical, categorical	Mean ± SD	<15, 15-200, >200

### Ethics Approval and Consent to Participate

Ethical clearance to conduct the study has been obtained from the Muhimbili University of Health and Allied Sciences and Research and Publication Ethical Committee. A formal written informed consent in Swahili was used for eligible individuals. Nonconsenting patients and those who were not eligible for the study were attended to by the clinical team per SCD management guidelines. Personal, clinical and laboratory information is being kept with utmost confidentiality in keeping with the standards and procedures guiding the organization.

## Results

Enrolment commenced in January 2015 and concluded in September 2016. During the study period, 513 SCD individuals were hospitalized, of these 387 (72.8%) fulfilled inclusion criteria and were enrolled. Final results of batched laboratory tests are expected in January 2018. Data analysis is expected to be done in February 2018. The study results will be published in a peer-reviewed journal.

## Discussion

### Study Rationale

Severe anemia is the most common cause of SCD morbidity and mortality in Tanzania. Although the clinical and laboratory factors associated with severe anemia have been described in the general population of sub-Saharan Africa [6,8,11,26,27], detailed characterization of these factors in SCD individuals is lacking.

Currently available data in the literature list possible causes of severe anemia in SCD cases. These include infections like malaria, bacteraemia and HIV infections as well as hookworm infestation [8-12,18]. Other factors include nutritional deficiencies like iron, vitamins A, B12 and folate [6,13]. Hyperhemolysis has also been shown to contribute to anemic crises [17]. Multiple transfusions increase the risk of hemolytic transfusion reactions, alloimmunization [23,24,26-28] and iron overload [29,30]. Furthermore, severe anemia in SCD patients has been shown to be an independent predictor of death [6].

This protocol describes in detail participant enrolment, and clinical and laboratory procedures. The study involved collecting multiple clinical and laboratory variables during the hospitalization period. No data was collected outside the hospitalization period. Drawing from 12 years' experience in clinical longitudinal surveillance, this protocol will contribute significantly to building a basis for a single-center, multivariable clinical study. It will also contribute towards harmonized inpatient clinical and laboratory procedures. The methodology will ensure the validity and reliability of data on the prevalence of severe anemia and associated factors in hospitalized SCD individuals.

### Limitations

This study has several limitations. First, its cross-sectional design precludes us from establishing a cause-effect relationship. There is also potential for patient recall bias, especially in establishing past medical history. In this instance patients were asked to recall events from 12 months prior to hospitalization. Second, although interventions, like the use of antibiotics and/or antimalarial drugs in the days prior to admission were recorded, we have no certain way of knowing if the information provided is accurate. MNH is a national referral hospital; most patients are referred from peripheral facilities where they would have received interventions. Third, in this study, we are using 3 different laboratories to carry out various tests. Although the standard operating procedures for laboratory procedures are present and enforced, we cannot entirely eliminate human errors. Fourth, by the fact that MNH is a tertiary institution, there is a simple selection bias. This methodology paper will provide a useful and practical model for conducting inpatient clinical cross-sectional studies that cover a wide range of clinical and laboratory variables. The results of this study will be published in a peer-reviewed journal and have a potential to guide the management of severe anemia in SCD in order to reduce associated morbidity and mortality.

## Abbreviations

ALT: alanine aminotransferase
AST: aspartate aminotransferase
CRF: case report form
CRP: C reactive protein
EDTA: ethylenediamine tetraacetic acid
Hb: hemoglobin
LDH: lactate dehydrogenase
MNH: Muhimbili National Hospital
MSC: Muhimbili Sickle Cohort
RDT: malaria rapid test
SCD: sickle cell disease
